# Axonal Protection by Tacrolimus with Inhibition of NFATc1 in TNF-Induced Optic Nerve Degeneration

**DOI:** 10.1007/s11064-019-02804-6

**Published:** 2019-05-13

**Authors:** Chihiro Tsukahara, Kana Sase, Naoki Fujita, Hitoshi Takagi, Yasushi Kitaoka

**Affiliations:** 1 Department of Ophthalmology, St. Marianna University School of Medicine, 2-16-1 Sugao, Miyamae-ku, Kawasaki, 216-8511 Kanagawa Japan; 2 Department of Molecular Neuroscience, St. Marianna University Graduate School of Medicine, Kawasaki, Japan

**Keywords:** Tacrolimus, Calcineurin inhibitor, Calcineurin, NFATc1, Tumor necrosis factor, Optic nerve

## Abstract

Tacrolimus, a calcineurin (CaN) inhibitor, has been used for treatment of refractory allergic ocular disease, although its role in optic nerve degeneration remains to be elucidated. In this study, we investigated whether tacrolimus modulates tumor necrosis factor (TNF)-mediated axonal degeneration and whether it alters nuclear factor of activated T cells (NFATc), a downstream effector of CaN signaling. Immunoblot analysis showed no significant difference in CaNAα protein levels in optic nerve on day 3, 7, or 14 after TNF injection compared with PBS injection. However, a significant increase in NFATc1 protein level was observed in optic nerve 7 days after TNF injection. This increase was negated by simultaneous administration of tacrolimus. Administration of tacrolimus alone did not change the NFATc1 protein level in comparison to that observed after PBS injection. A significant increase in TNF protein level was observed in optic nerve 14 days after TNF injection and this increase was prevented by tacrolimus. Immunohistochemical analysis showed the immunoreactivity of NFATc1 to be increased in optic nerve after TNF injection. This increased immunoreactivity was colocalized with glial fibrillary acidic protein and was suppressed by tacrolimus. Treatment of tacrolimus significantly ameliorated the TNF-mediated axonal loss. These results suggest that tacrolimus is neuroprotective against axon loss in TNF-induced optic neuropathy and that the effect arises from suppression of the CaN/NFATc1 pathway.

**Electronic supplementary material:**

The online version of this article (10.1007/s11064-019-02804-6) contains supplementary material, which is available to authorized users.

## Introduction

Glaucoma is, in developed countries, one of the most common diseases that can lead to irreversible blindness. It is characterized by a slowly progressive retinal ganglion cell (RGC) pathology in which axonal degeneration of RGCs in the optic nerve precedes death of the RGC body. The only reliable clinical treatment for glaucoma is lowering intraocular pressure. However, means of neuroprotection are being studied as a complementary treatments.

Calcineurin (CaN) is a serine/threonine phosphatase, dependent on Ca^2+^/calmodulin (CaM). It is composed of two subunits, CaNA and CaNB. CaNA which has a CaM-binding domain, acts as a 60-kDa catalytic subunit. CaNB, a 19-kDa regulatory subunit, has a Ca^2+^-binding domain [1, 2]. There are three isoforms (α, β, γ) of CaNA, with CaNAγ expression limited to testis and CaNAα and CaNAβ expressed in all tissues, but in varying ratios. In brain, for example, CaNAα is more abundant than CaNAβ in brain [3, 4].

Increased intracellular Ca^2+^ activates CaN, and the CaN combines with the Ca^2+^/CaM, leading to dephosphorylation of nuclear factor of activated T cells (NFATc) in the cytoplasm. Dephosphorylated NFATc translocates to the nucleus to regulate the transcription of cytokines such as interleukin (IL)-2 and tumor necrosis factor (TNF) [5, 6]. There are 4 Ca^2+^/CaM-dependent NFATc transcription factors: NFATc1 (NFAT2), NFATc2 (NFAT1), NFATc3 (NFAT4), and NFATc4 (NFAT3) [7]. All are expressed in human brain, with NFATc1 reported to be present in astrocytes [8]. Increased NFATc1 activity has been observed in the cerebral cortex in a rat traumatic brain injury model [9]

Tacrolimus, a CaN inhibitor, forms a complex with immunophilin (FKBP) and inhibits CaN enzyme activity [10]. Tacrolimus has been clinically applied as an immunosuppressant and also applied via eyedrops for severe allergic conjunctivitis. In addition, tacrolimus has been shown to have a protective effect on RGCs in an optic nerve crush model [11, 12] and both on RGCs and optic nerve axons in an elevated intraocular pressure (IOP) model [13], although its mechanism remains to be elucidated.

TNF is a cytokine that is synthesized and released from microglia and astrocytes around several neurons. An association between TNF-related neurotoxicity and glaucomatous optic neuropathy has been reported [14]. For example, a previous study has shown that ratio of TNF positive patients in aqueous humor was significantly higher in glaucoma group than cataract group [15]. In addition, a study from human glaucomatous retina has shown a significant up-regulation of TNF/TNF receptor 1 signaling [16]. More recently, a TNF antagonist was shown to reduce RGC hyperexcitability in a rat hypertensive glaucoma model [17]. We previously reported that intravitreal injection of TNF induces optic nerve degeneration in rats, in turn inducing RGC loss in rats [18]. In the present study, we tested whether tacrolimus modulates TNF-mediated axonal loss and it alters CaN/NFATc1 signaling.

## Materials and Methods

### Animals

For experiments, male Wistar rats of 50 to 55 days old were used. All experiments were conducted in accordance with statements of the Association for Research in Vision and Ophthalmology statement on the Use of Animals in Ophthalmic and Vision Research. Animals were kept in a controlled condition with a temperature of 23 ± 1 °C, humidity of 55 ± 5%, and light between 6 am and 6 pm.

### TNF Injection Model

Intravitreal injection of TNF (Sigma-Aldrich, St Louis, MO) or tacrolimus (Senju pharmaceutical, Osaka, Japan) was performed as previously described [19]. Briefly, intramuscular injection anesthesia with mixture of ketamine-xylazine (10 mg/kg and 4 mg/kg respectively) was administered to rats. A single 2-μL injection of 10 ng TNF in 0.01 M phosphate-buffered saline (PBS; pH 7. 40) was administered intravitreally into the animal’s right eye under a microscope. PBS alone, as a control, was administered to the left eye. Three, seven or fourteen days after the intravitreal injections, the rats were killed by an overdose of sodium pentobarbital administered intraperitoneally, and the eyes were subsequently enucleated.

### Immunoblot Analysis

One hundred and two rats were used for immunoblot analysis as described previously [19]. Briefly, optic nerves (4 mm in length) and retinas were collected, homogenized, and centrifuged at 15,000×*g* for 15 min at 4 °C after 3, 7 or 14 days after the intravitreal injections. Optic nerve specimens (n = 2) were collected for each sample. Each retina was used as one sample. Protein concentrations were measured with the Bio-Rad Protein Assay kit (Bio-Rad, Hercules, CA). Protein samples (3 μg per lane of optic nerve sample; 5 μg per lane of retina) were subjected to SDS-PAGE on 10% polyacrylamide gels and transferred to an enhanced chemiluminescent membrane (EMD Millipore Corporation, Temecula, CA). Tris buffered saline-0.1% Tween-20 (T-TBS) containing 5% skim milk was used for blocking of membranes. Membranes were reacted with CaNAα antibody (1:200; EMD Millipore Corporation), NFATc1 antibody (1:200; Santa Cruz Biotechnology, TX), TNF antibody (1:200; R&D Systems, MN) or anti-β-actin antibody (1:500; Sigma-Aldrich) in T-TBS. The membranes were then reacted to peroxidase-labeled anti-rabbit secondary antibody (MP Biomedicals, Solon, OH), peroxidase-labeled anti-goat secondary antibody (MP Biochemicals) or peroxidase-labeled anti-mouse secondary antibody (MP Biochemicals) diluted 1:5000 in T-TBS. Immunoblots were detected by a chemiluminescence membrane detection system (ECL Plus Western Blotting Detection Reagents; Amersham Pharmacia Biotech).

### Immunohistochemical Analysis

Twelve rats were used for immunohistochemical analysis as described previously [19]. After injection of PBS, TNF or TNF with tacrolimus, eyes were put into 4% paraformaldehyde. After paraffin sections were made, sections were incubated with anti-NFATc1 antibody (1:200; Santa Cruz Biotechnology), anti-glial fibrillary acidic protein (GFAP) antibody (1:200; Dako, Tokyo, Japan) or anti ionized calcium-binding adaptor molecule1 (Iba-1; a marker of microglial cells) antibody (1:100; WAKO, Osaka, Japan). After washing, the sections were reacted with rhodamine-labeled or FITC-labeled secondary antibodies (1:100; Cappel, Aurora, OH) in the dark. After several washing, the sections were mounted on slides in DAPI-containing medium (Vectashield with DAPI; Vector Laboratories, Burlingame, CA). BSA was dropped as a negative control by replacing the primary antibody.

### Axon Counting in Optic Nerve

Morphometric analysis of each optic nerve in samples from 23 rats was performed as described previously [19]. Two weeks after the intravitreal injections, eyes were obtained from the animals. The optic nerves, 4-mm segments, were obtained from 1 mm behind the globe. The segments were soaked in Karnovsky’s solution for 24 h at 4℃, processed and embedded in acrylic resin. Cross Sections (1 μm thick) were cut and stained with a solution of 1% paraphenylene-diamine (Sigma-Aldrich) in absolute methanol. For each slice, images at the center and at each quadrant of the periphery (approximately 141.4 μm from the center) were obtained with a light microscope (BX51, Olympus, Tokyo, Japan), which was equipped with a 100 × coupled digital camera (MP5Mc/OL, Olympus) and QCapture Pro software (version 5.1, QImaging, Surrey, Canada). Aphelion image processing software (Version 3.2, ADCIS, Hérouville Saint-Clair, France) quantified the acquired images. The number of axons was calculated in five distinct regions from each eye, 1446.5 μm^2^ each (each quadrant in the periphery plus the center; total area of 7232.3 μm^2^ per eye). An average of the number of axons per eye was calculated, and this average value was calculated as a number per square millimeter. For each experimental condition at least 5 eyes were used for the analysis.

### Statistical Analysis

Data are presented as mean ± SEM. Differences between groups were analyzed by one-way ANOVA and then Dunnett’s test. A probability value (*p* value) of < 0.05 was considered statistically significant.

## Results

### CaNAα Protein Levels in the Optic Nerve After TNF Injection

**Fig. 1:**
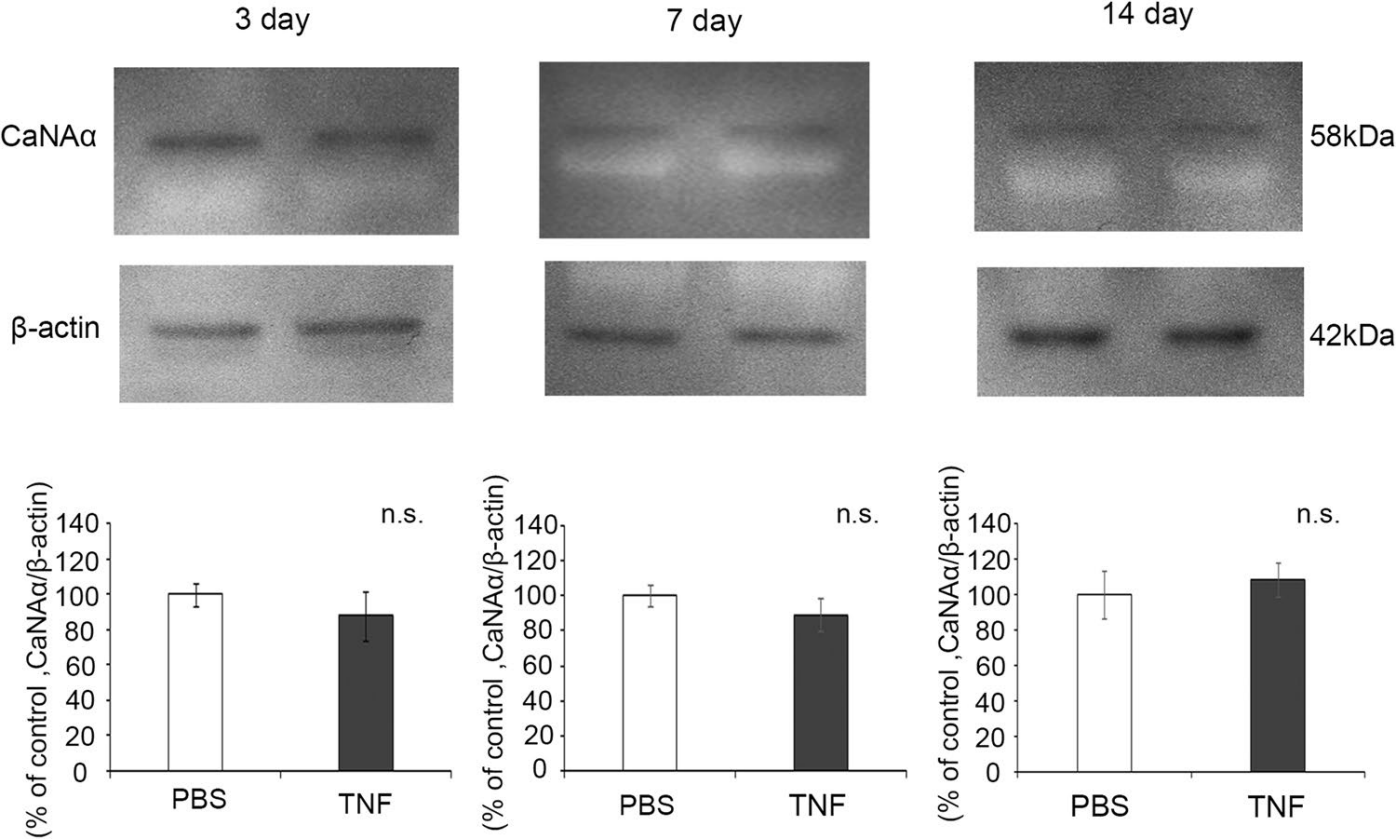
CaNAα protein expression in optic nerves on days 3, 7 and 14 after intravitreal injection of PBS or TNF. Immunoblotting values are normalized to β-actin. Values are expressed as percentages of control and represent mean ± SEM.* n* = 4

Immunoblot analysis showed a band of CaNAα at 58 kDa in the optic nerve sample, and no time point did the CaNAα protein level after TNF injection differ from that after PBS injection. No cleaved CaNAα was observed in the optic nerve at any time point after TNF injection (Fig. 1).

### NFATc1 Protein Levels in the Optic Nerve After TNF Injection

**Fig. 2:**
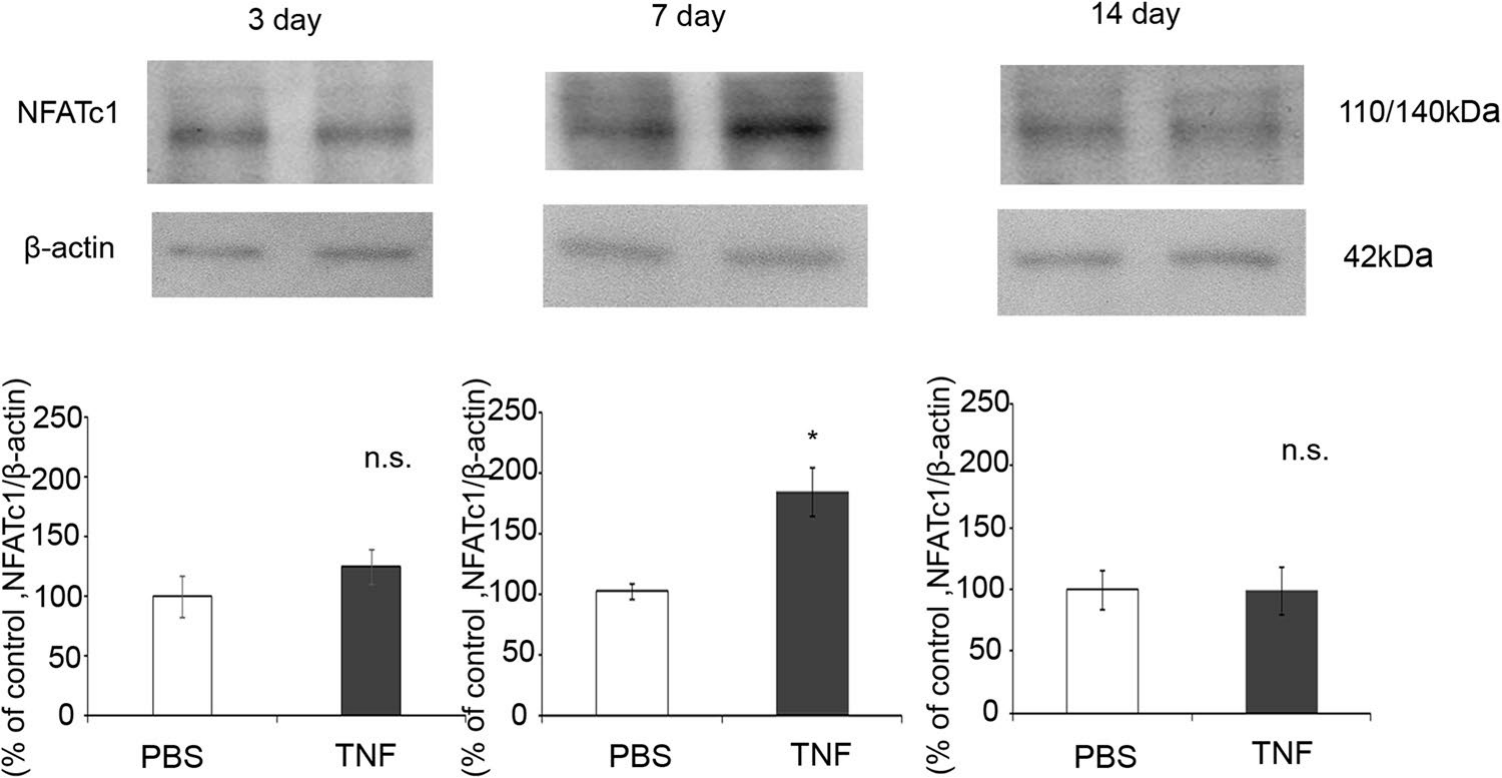
NFATc1 protein expression in optic nerves on days 3, 7 and 14 after intravitreal injection of PBS or TNF. Immunoblotting values are normalized to β-actin. Values are expressed as percentages of control and represent mean ± SEM.* n* = 4. *p < 0.05

Immunoblot analysis showed a band of NFATc1 at 110/140 kDa in the optic nerve sample. NFATc1 protein level was significantly increased in optic nerve 7 days after TNF injection (Fig. 2). However, there were no statistically significant changes in NFATc1 protein levels 3 days and 14 days after TNF injection compared with the control (Fig. 2).

### Effect of Tacrolimus on NFTATc1 Protein Level in Optic Nerve

**Fig. 3:**
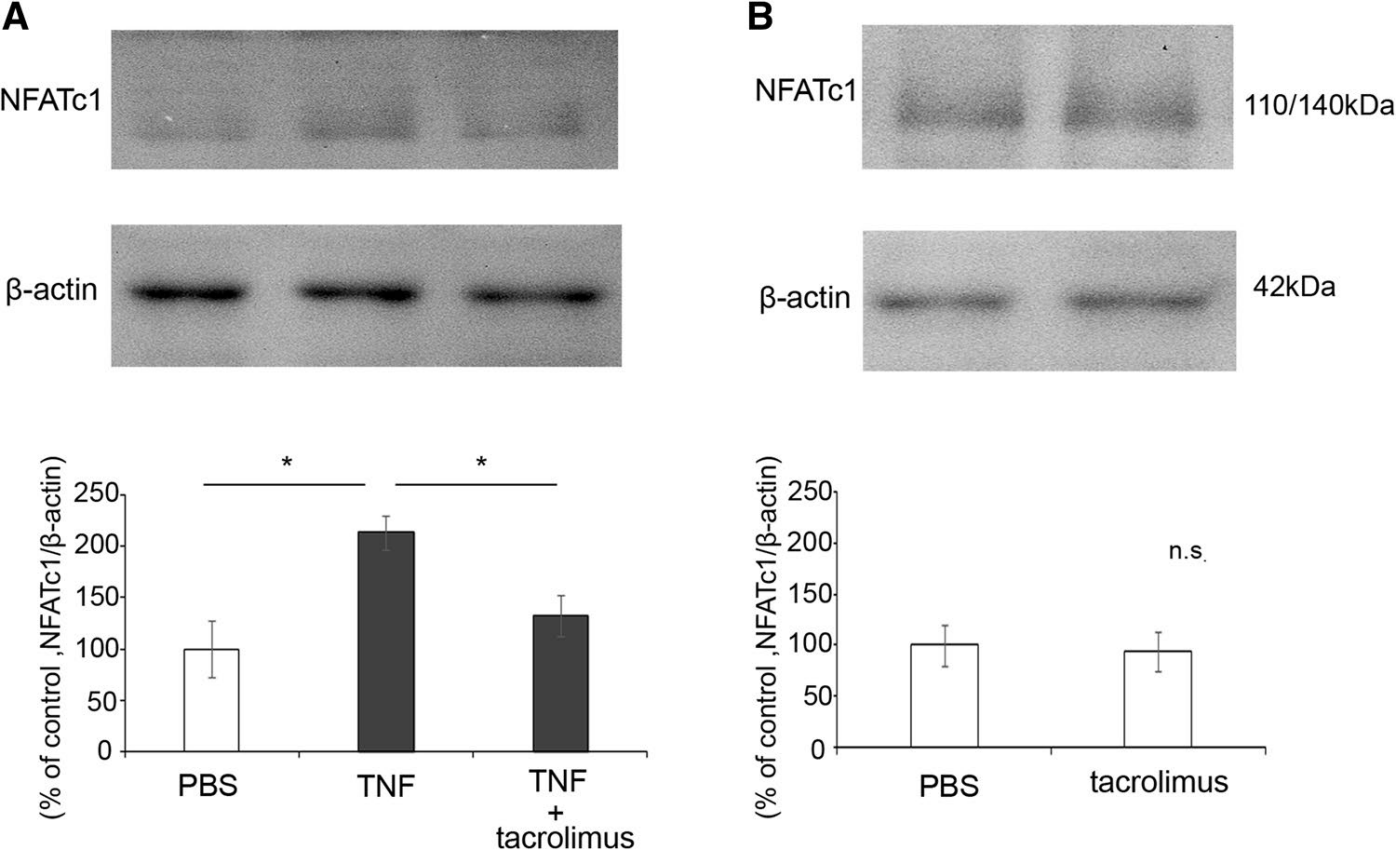
NFATc1 protein expression in optic nerves 7 days after intravitreal injection. Immunoblotting values are normalized to β-actin. **a** NFATc1 expression after injection of PBS, TNF, or TNF + 10^−4^ M tacrolimus. Values are expressed as percentages of control and represent mean ± SEM.* n* = 4. *p < 0.05. **b** NFATc1 expression after injection of PBS or 10^−4^ M tacrolimus. Values are expressed as percentages of control and represent mean ± SEM. *n* = 4

Because the NFATc1 protein level was shown to be significantly increased in the optic nerve 7 days after TNF injection, we examined the effect of tacrolimus on the NFATc1 levels at this time point. The TNF-induced increase in NFATc1 was negated by simultaneous administration of tacrolimus (Fig. 3a). There was no significant difference in NFATc1 protein expression after tacrolimus treatment alone compared with PBS administration (Fig. 3b).

### TNF Protein Levels in the Optic Nerve After TNF Injection

**Fig. 4:**
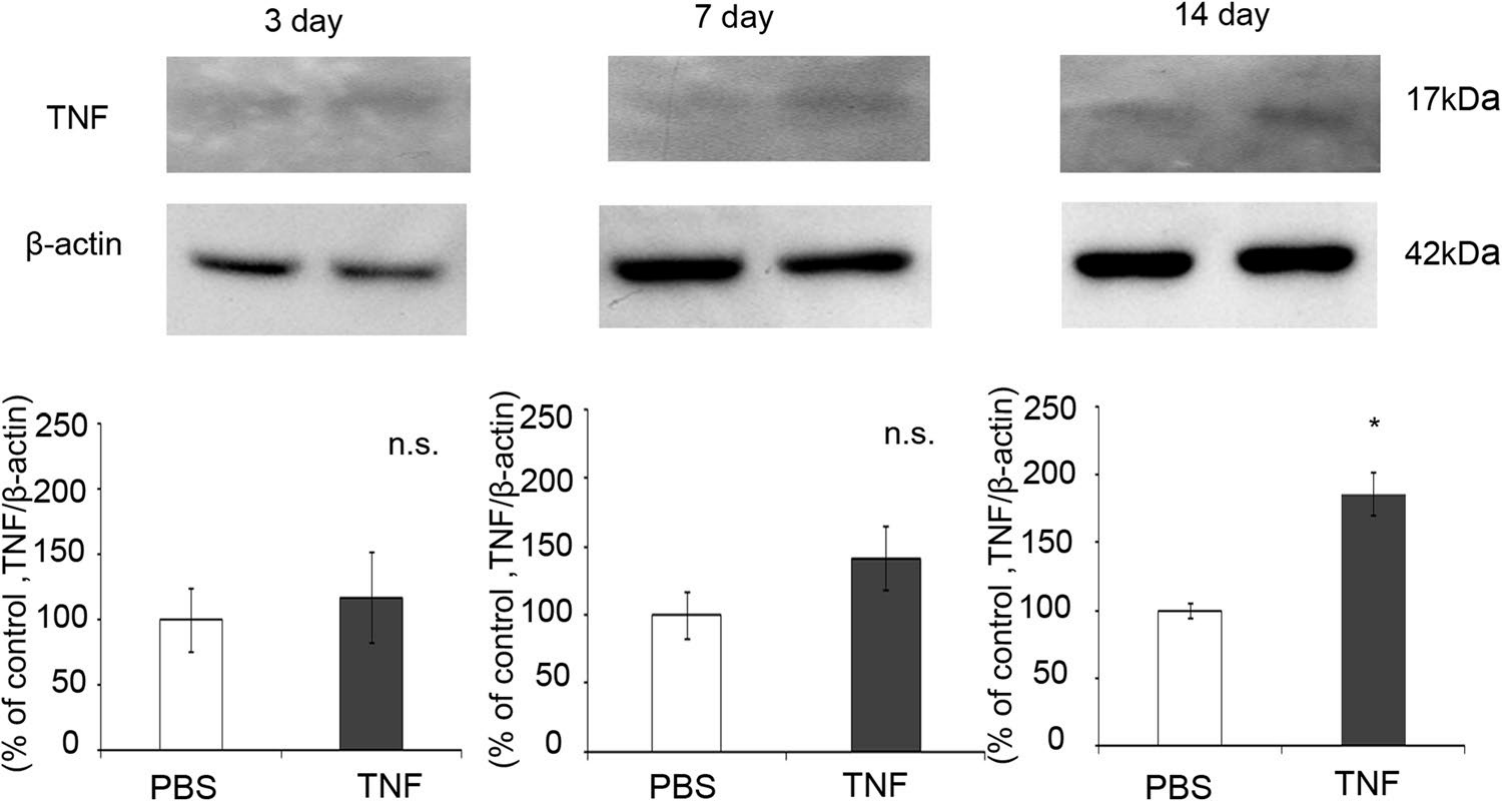
TNF protein expression in optic nerves on days 3, 7 and 14 after intravitreal injection of PBS or TNF. Immunoblotting values are normalized to β-actin. Values are expressed as percentages of control and represent mean ± SEM.* n* = 3. *p < 0.05

Immunoblot analysis showed a faint band of TNF at 17 kDa in the optic nerve sample after PBS injection (Fig. 4). There was no significant change in TNF protein level 3 days after TNF injection compared with the control. Although TNF protein level tended to increase 7 days after TNF injection, it was not statistically significant (Fig. 4). However, there was a significant increase in TNF protein level 14 days after TNF injection compared with the control (Fig. 4).

### Effect of Tacrolimus on TNF Protein Level in Optic Nerve

**Fig. 5:**
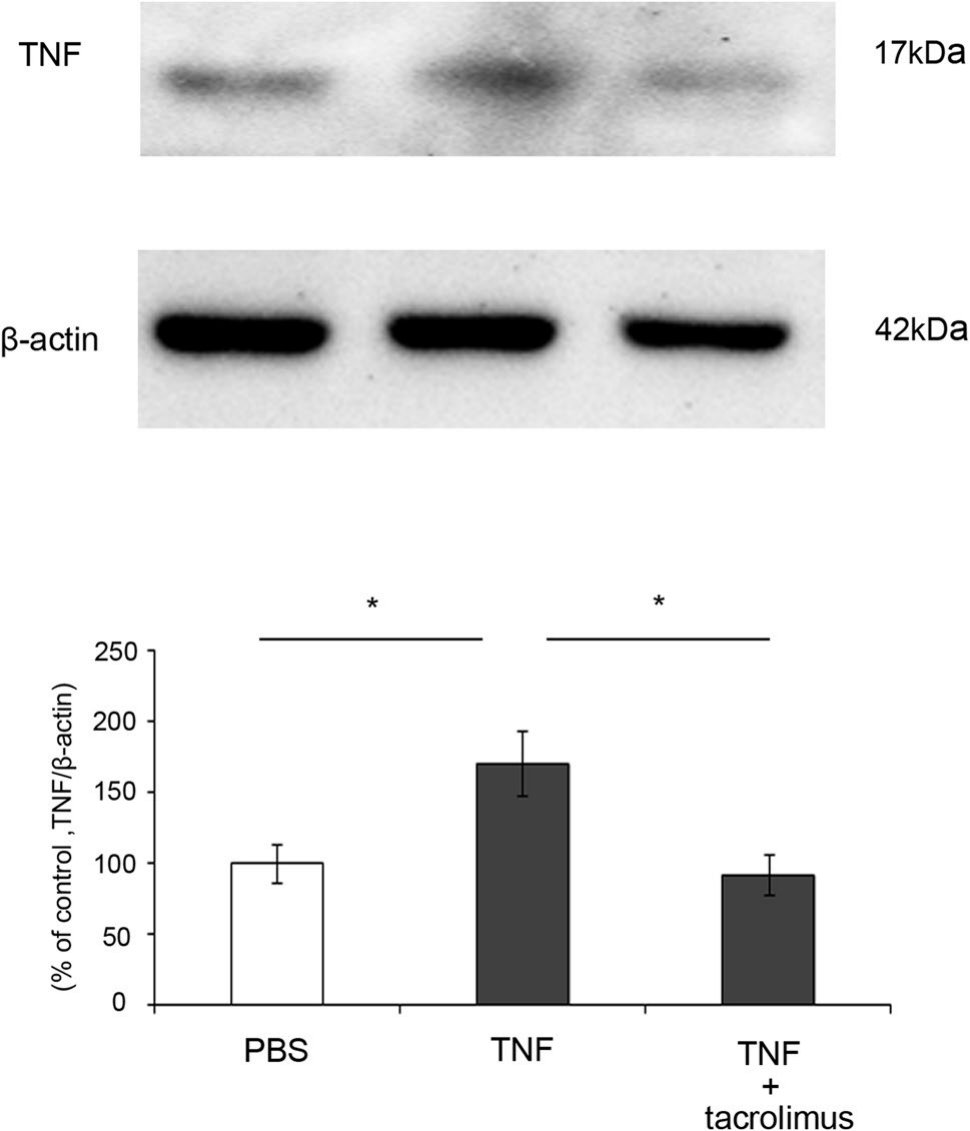
TNF protein expression in optic nerves 14 days after intravitreal injection. Immunoblotting values are normalized to β-actin. TNF expression after injection of PBS, TNF, or TNF + 10^−4^ M tacrolimus. Values are expressed as percentages of control and represent mean ± SEM.* n* = 3. *p < 0.05

Because the TNF protein level was shown to be significantly increased in the optic nerve 14 days after TNF injection, we examined the effect of tacrolimus on the TNF levels at this time point. The TNF-induced increase in TNF protein level was negated by simultaneous administration of tacrolimus (Fig. 5).

### Localization of NFATc1 in Optic Nerve

**Fig. 6:**
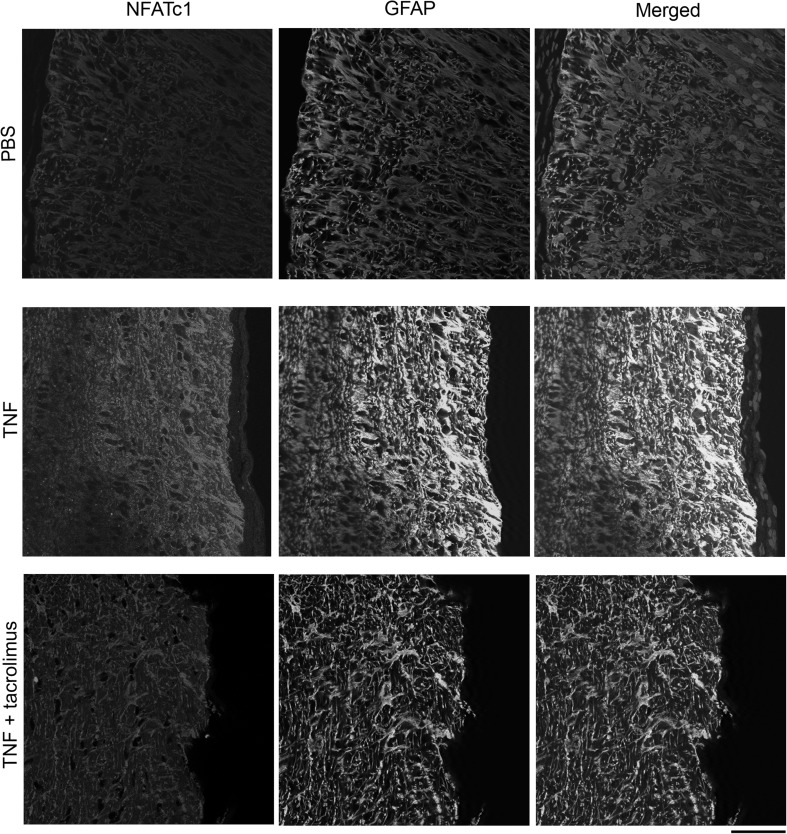
Immunohistochemistry for NFATc1 in longitudinal sectioned optic nerves. NFATc1 immunoreactivity increased after TNF injection. Apparent colocalization of NFATc1 with GFAP was seen in the TNF group. The increased immunoreactivity was suppressed by tacrolimus. Scale bar = 50 µm

**Fig. 7:**
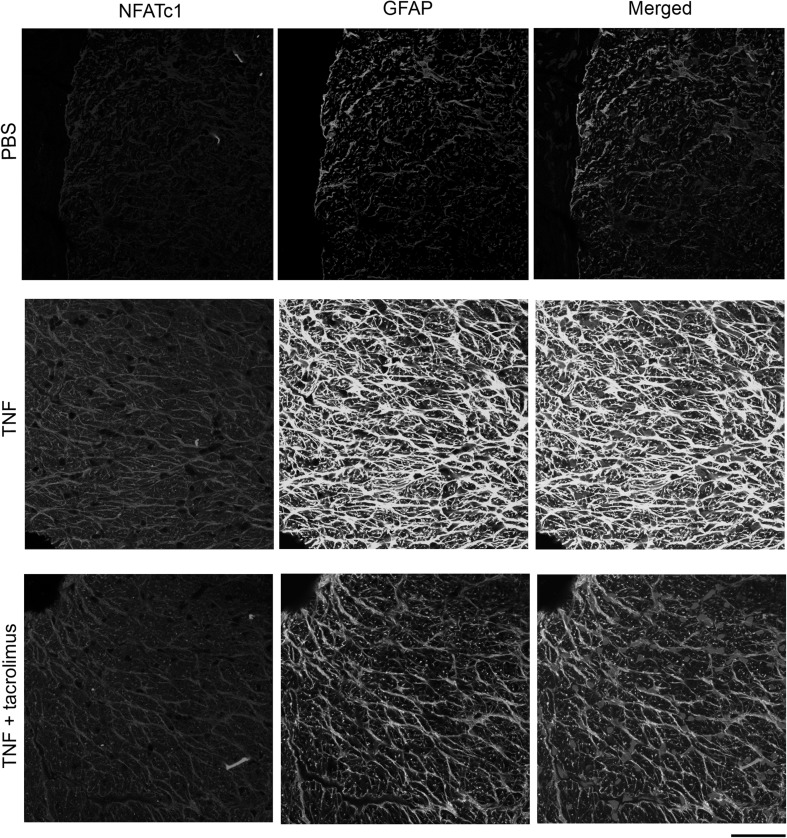
Immunohistochemistry for NFATc1 in cross sectioned optic nerves. NFATc1 immunoreactivity increased after TNF injection. Apparent colocalization of NFATc1 with GFAP was seen in the TNF group. The increased immunoreactivity was suppressed by tacrolimus. Scale bar = 50 µm

Upon immunohistochemical analysis, faint NFATc1 immunoreactivity was observed in longitudinal sectioned optic nerve after PBS injection, whereas increased NFATc1 immunoreactivity was observed 7 days after TNF injection (Fig. 6). This increased immunoreactivity was colocalized with GFAP, a marker of astrocyte (Fig. 6), and was suppressed by tacrolimus (Fig. 6). Similar findings were observed in cross sectioned optic nerves. The immunoreactivity of NFATc1 was increased by TNF which is colocalized with GFAP, and this was suppressed by tacrolimus (Fig. 7).

### Tacrolimus Prevented TNF-Induced Optic Nerve Degeneration

**Fig. 8:**
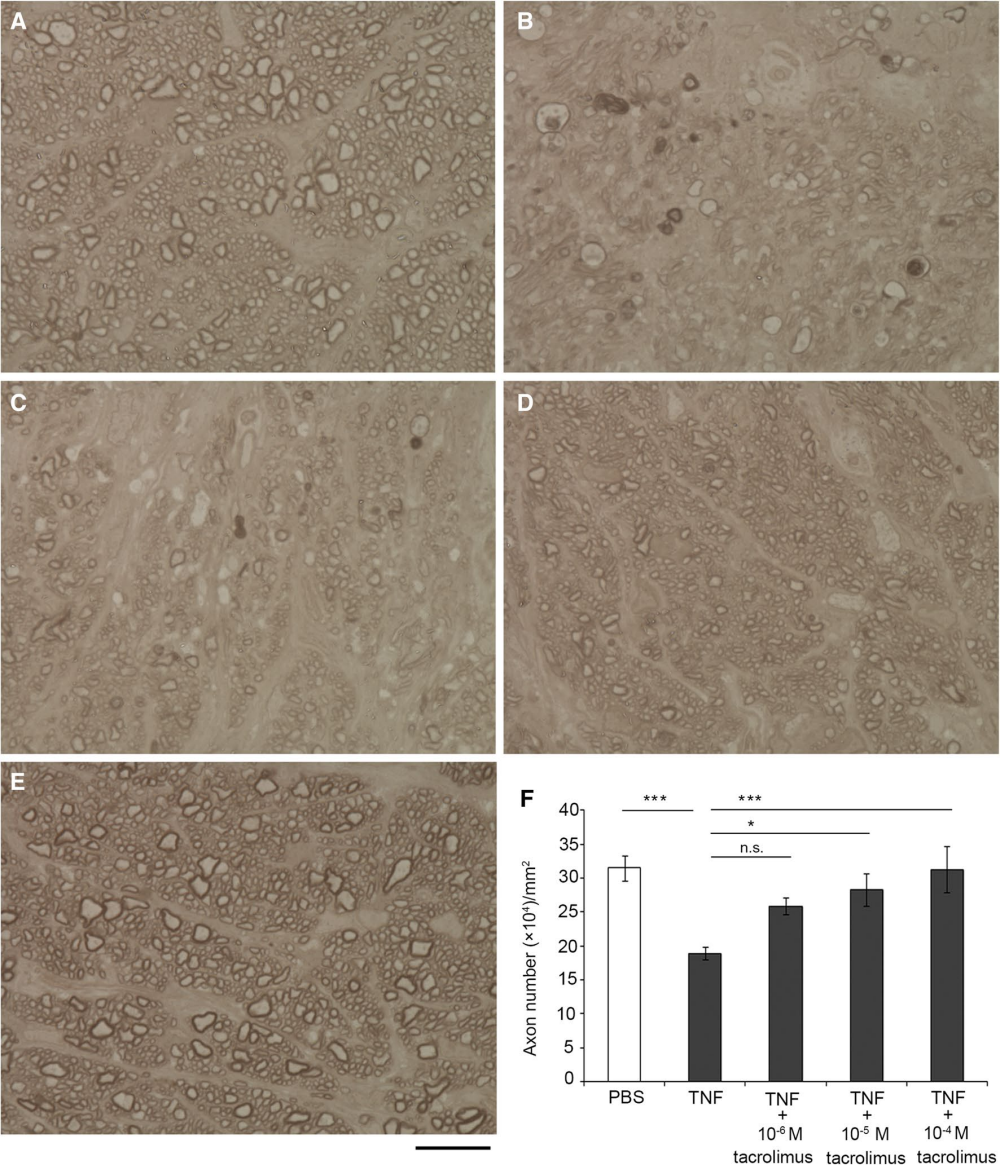
Tacrolimus exerted axonal protection in optic nerve. Histological findings 14 days following injection of PBS (**a**), TNF (**b**), TNF + 10^−6^ M tacrolimus (**c**), TNF + 10^−5^ M tacrolimus (**d**), or TNF + 10^−4^ M tacrolimus (**e**). Scale bar = 10 μm. **f** Comparison of axon numbers. *n* = 5–6 per group. *p < 0.05, ***p < 0.005

We confirmed degeneration in the optic nerve axons 14 days after TNF injection (Fig. 8b) compared with PBS injection (Fig. 8a) *(p* = 0.0015). Although treatment of 10^−6^ M tacrolimus had a certain axonal protective effect (Fig. 8c), the neurodegeneration induced by TNF was noticeable. In addition, the protective effect was not statistically significant when the number of axons was compared (*p* = 0.0684). However, treatment with 10^−5^ M tacrolimus (Fig. 8d) and 10^−4^ M tacrolimus (Fig. 8e) yielded substantial axonal protection. Quantitative analysis showed statistically significant protections (Fig. 8f, TNF vs. TNF + 10^−5^ M tacrolimus, *p* = 0.0146; TNF vs. TNF + 10^−4^ M tacrolimus, *p* = 0.0013). These protective effects were shown to appear in a concentration-dependent manner.

## Discussion

The present study demonstrated a significant increase in NFATc1 expression in optic nerves after TNF injection. NFATc1 is present in glial cells in optic nerve. Tacrolimus prevented the increase in NFATc1 expression and ameliorated the loss of axons of the optic nerve after TNF injection. These results suggest that the protective effect of tacrolimus may be involved in its inhibitory effect on NFATc1 expression.

In a previously reported study, cleaved CaN was found in retinal protein in the rat IOP elevation model but not in retinal protein in the optic nerve crush model [13]. In our current study, cleaved CaN was not observed in retinal protein (Supplementary Fig. 1) or in optic nerve protein (Fig. 1) after TNF injection. Under physiological elevation of intracellular Ca^2+^, CaNA activity is reversible. However, under a pathophysiological condition such as kainate- or glutamate-induced excitotoxicity [2] or cerebral ischemia [20], which causes excessive intracellular Ca^2+^ influx, CaNA activity is lasting. Calpain, a Ca/CaM dependent protein, puts CaN into a constitutively activated state by cleaving the self-suppressing region of CaNA [2]. However, it is unclear why only IOP elevation caused cleavage of CaN but that optic nerve crush and TNF injection model did not. One hypothesis posits that the increased intracellular Ca^2+^ concentration is limited in the TNF model and that the increase is not enough to occur CaN cleavage or alter the CaN protein level. Nonetheless, we cannot deny the possibility of CaN activation in the TNF model because lack of agreement between the protein levels and the enzyme activity of CaN has been reported [21].

Recent studies have reported NFATc3 expression in human lamina cribrosa cells [22] and NFATc1 expression in human trabecular meshwork cells [23]. Our study appears to be the first to reveal expression of NFATc1 in optic nerve. The protein levels of NFATc1 in optic nerve increased with TNF-induced optic nerve degeneration. Increased expression of NFAT4 has been reported in activated astrocytes throughout the axon/dendrite layers of CA1 and the dentate gyrus [24]. It has also been reported that in quiescent astrocytes, TNF recruits CaN to stimulate a canonical NFAT pathway [25]. We found NFATc1 immunoreactivity colocalized with GFAP immunoreactivity but not with Iba1 immunoreactivity (Supplementary Fig. 2), indicating that NFATc1 is present in astrocytes not in microglial cells in optic nerve. It is interesting to note that Ca-channel agonist increases TNF via the CaN/NFAT system in PC12 cells [26]. Therefore, it is possible that increased Ca^2+^ may result in CaN/NFAT activation, thereby increasing in TNF in certain cell death. Consistent with this idea, the present study demonstrated that increased NFATc1 protein level was observed 7 days and increased TNF protein level was observed 14 days after TNF injection, suggesting that NFATc1 exists upstream of TNF. It was reported that NFATc1β, an isoform of NFATc1, upregulated TNF production in NIH3T3 cells [27]. On the other hand, other possibility is that there are some compensatory effects by the other NFATc proteins, because it was demonstrated that TNF upregulation was the main cause of cell death controlled by NFATc2 in NIH3T3 cells [28].

In the present study, we found that tacrolimus significantly prevented the TNF-induced increase in NFATc1 expression in optic nerve. Morphometric analysis revealed that tacrolimus significantly prevented TNF-induced axon loss. These findings are consistent with those of a previously reported study demonstrating that tacrolimus attenuates the activation of astrocytes and microglia of brain in a rat cerebral ischemia model [29]. That study also demonstrated that tacrolimus attenuates the expression of cytokines, such as TNF-α, in glia after cerebral ischemia [29]. The present study also found that tacrolimus abolished increment of TNF protein level in optic nerve. Moreover, it was reported that tacrolimus abolished NFAT activation in the anteroventral cochlear nucleus neurons of mice [30]. Furthermore, it was shown that increased expression of NFAT4 in activated astrocytes was observed in hippocampus of a murine Alzheimer's disease model [31] and that amyloid β expression in hippocampus was suppressed by administration of tacrolimus or NFAT inhibitor in the murine Alzheimer's disease model [32]. Thus, inhibition of NFAT by tacrolimus may have beneficial effects against several types of neuronal damages. Similarly to the results of immunoblotting, the present immunohistochemical study showed that the expression of NFATc1 tended to increase after TNF injection, and this increase was suppressed by tacrolimus. Increased expression of GFAP induced by TNF tended to be suppressed by tacrolimus. Intravitreal injection of TNF may lead to activation of microglia and astrocytes in optic nerve [18, 33]. Therefore, it is reasonable to speculate that tacrolimus suppresses the activation of glial cells and inhibits the positive feedback loop among activated glia, cytokines and CaN/NFAT [34].

In conclusion, tacrolimus may protect against axon loss in TNF-induced optic neuropathy by inhibiting the CaN/NFATc1 pathway.

## Electronic supplementary material

Below is the link to the electronic supplementary material.

Supplementary material 1 (TIFF 2709 kb)

Supplementary material 2 (TIFF 5688 kb)

## References

[1] Klee CB, Ren H, Wang X (1998). Regulation of the calmodulin-stimulated protein phosphatase, calcineurin. J Biol Chem.

[2] Wu HY, Tomizawa K, Oda Y, Wei FY, Lu YF, Matsushita M, Li ST, Moriwaki A, Matsui H (2004). Critical role of calpain-mediated cleavage of calcineurin in excitotoxic neurodegeneration. J Biol Chem.

[3] Perrino BA, Wilson AJ, Ellison P, Clapp LH (2002). Substrate selectivity and sensitivity to inhibition by FK506 and cyclosporin A of calcineurin heterodimers composed of the a or b catalytic subunit. Eur J Biochem.

[4] Kuno T, Mukai H, Ito A, Chang CD, Kishima K, Saito N, Tanaka C (1992). Distinct cellular expression of calcineurin A alpha and A beta in rat brain. J Neurochem.

[5] Reppert S, Zinser E, Holzinger C, Sandrock L, Koch S, Finotto S (2015). NFATc1 deficiency in T cells protects mice from experimental autoimmune encephalomyelitis. Eur J Immunol.

[6] Serrano-Pérez MC, Martín ED, Vaquero CF, Azcoitia I, Calvo S, Cano E, Tranque P (2011). Response of transcription factor NFATc3 to excitotoxic and traumatic brain insults: identification of a subpopulation of reactive astrocytes. Glia.

[7] Hogan PG, Chen L, Nardone J, Rao A (2003). Transcriptional regulation by calcium, calcineurin, and NFAT. Genes Dev.

[8] Pérez-Ortiz JM, Serrano-Pérez MC, Pastor MD, Martín ED, Calvo S, Rincón M, Tranque P (2008). Mechanical lesion activates newly identified NFATc1 in primary astrocytes: implication of ATP and purinergic receptors. Eur J Neurosci.

[9] Wu Q, Liu G, Xu L, Wen X, Cai Y, Fan W, Yao X, Huang H, Li Q (2016). Repair of neurological function in response to FK506 through CaN/NFATc1 pathway following traumatic brain injury in rats. Neurochem Res.

[10] Liu J, Farmer JD, Lane WS, Friedman J, Weissman I, Schreiber SL (1991). Calcineurin is a common target of cyclophilin-cyclosporin A and FKBP-FK506 complexes. Cell.

[11] Freeman EE, Grosskreutz CL (2000). The effects of FK506 on retinal ganglion cells after optic nerve crush. Invest Ophthalmol Vis Sci.

[12] Rosenstiel P, Schramm P, Isenmann S, Brecht S, Eickmeier C, Bürger E, Herdegen T, Sievers J, Lucius R (2003). Differential effects of immunophilin-ligands (FK506 and V-10,367) on survival and regeneration of rat retinal ganglion cells in vitro and after optic nerve crush in vivo. J Neurotrauma.

[13] Huang W, Fileta JB, Dobberfuhl A, Filippopolous T, Guo Y, Kwon G, Grosskreutz CL (2005). Calcineurin cleavage is triggered by elevated intraocular pressure, and calcineurin inhibition blocks retinal ganglion cell death in experimental glaucoma. Proc Natl Acad Sci USA.

[14] Tezel G (2008). TNF-alpha signaling in glaucomatous neurodegeneration. Prog Brain Res.

[15] Sawada H, Fukuchi T, Tanaka T, Abe H (2010). Tumor necrosis factor-alpha concentrations in the aqueous humor of patients with glaucoma. Invest Ophthalmol Vis Sci.

[16] Yang X, Luo C, Cai J, Powell DW, Yu D, Kuehn MH, Tezel G (2011). Neurodegenerative and inflammatory pathway components linked to TNF-α/TNFR1 signaling in the glaucomatous human retina. Invest Ophthalmol Vis Sci.

[17] Zhao Y, Li Q, Li XY, Cui P, Gao F, Zhu K, Li LZ, Sun XH, Wang Z (2018). Involvement of mGluR I in EphB/ephrinB reverse signaling activation induced retinal ganglion cell apoptosis in a rat chronic hypertension model. Brain Res.

[18] Kitaoka Y, Kitaoka Y, Kwong JM, Ross-Cisneros FN, Wang J, Tsai RK, Sadun AA, Lam TT (2006). TNF-alpha-induced optic nerve degeneration and nuclear factor-kappaB p65. Invest Ophthalmol Vis Sci.

[19] Kitaoka Y, Sase K, Tsukahara C, Kojima K, Shiono A, Kogo J, Tokuda N, Takagi H (2017). Axonal protection by ripasudil, a Rho kinase inhibitor, via modulating autophagy in TNF-Induced optic nerve degeneration. Invest Ophthalmol Vis Sci.

[20] Shioda N, Han F, Moriguchi S, Fukunaga K (2007). Constitutively active calcineurin mediates delayed neuronal death through Fas-ligand expression via activation of NFAT and FKHR transcriptional activities in mouse brain ischemia. J Neurochem.

[21] Morioka M, Fukunaga K, Hasegawa S, Okamura A, Korematsu K, Kai Y, Hamada J, Nagahiro S, Miyamoto E, Ushio Y (1999). Activities of calcineurin and phosphatase 2A in the hippocampus after transient forebrain ischemia. Brain Res.

[22] Irnaten M, Zhdanov A, Brennan D, Crotty T, Clark A, Papkovsky D, O'Brien C (2018). Activation of the NFAT-calcium signaling pathway in human lamina cribrosa cells in glaucoma. Invest Ophthalmol Vis Sci.

[23] Faralli JA, Clark RW, Filla MS, Peters DM (2015). NFATc1 activity regulates the expression of myocilin induced by dexamethasone. Exp Eye Res.

[24] Furman JL, Sompol P, Kraner SD, Pleiss MM, Putman EJ, Dunkerson J, Mohmmad Abdul H, Roberts KN, Scheff SW, Norris CM (2016). Blockade of astrocytic calcineurin/NFAT signaling helps to normalize hippocampal synaptic function and plasticity in a rat model of traumatic brain injury. J Neurosci.

[25] Fernandez AM, Fernandez S, Carrero P, Garcia-Garcia M, Torres-Aleman I (2007). Calcineurin in reactive astrocytes plays a key role in the interplay between proinflammatory and anti-inflammatory signals. J Neurosci.

[26] Canellada A, Cano E, Sánchez-Ruiloba L, Zafra F, Redondo JM (2006). Calcium-dependent expression of TNF-alpha in neural cells is mediated by the calcineurin/NFAT pathway. Mol Cell Neurosci.

[27] Lucena PI, Faget DV, Pachulec E, Robaina MC, Klumb CE, Robbs BK, Viola JP (2016). NFAT2 isoforms differentially regulate gene expression, cell death, and transformation through alternative N-Terminal domains. Mol Cell Biol.

[28] Robbs BK, Lucena PI, Viola JP (2013). The transcription factor NFAT1 induces apoptosis through cooperation with Ras/Raf/MEK/ERK pathway and upregulation of TNF-α expression. Biochim Biophys Acta.

[29] Zawadzka M, Kaminska B (2005). A novel mechanism of FK506-mediated neuroprotection: downregulation of cytokine expression in glial cells. Glia.

[30] Luoma JI, Zirpel L (2008). Deafferentation-induced activation of NFAT (nuclear factor of activated T-cells) in cochlear nucleus neurons during a developmental critical period: a role for NFATc4-dependent apoptosis in the CNS. J Neurosci.

[31] Sompol P, Furman JL, Pleiss MM, Kraner SD, Artiushin IA, Batten SR, Quintero JE, Simmerman LA, Beckett TL, Lovell MA, Murphy MP, Gerhardt GA, Norris CM (2017). Calcineurin/NFAT signaling in activated astrocytes drives network hyperexcitability in Aβ-bearing mice. J Neurosci.

[32] Rojanathammanee L, Floden AM, Manocha GD, Combs CK (2015). Attenuation of microglial activation in a mouse model of Alzheimer's disease via NFAT inhibition. J Neuroinflammation.

[33] Kojima K, Kitaoka Y, Munemasa Y, Ueno S (2012). Axonal protection via modulation of the amyloidogenic pathway in tumor necrosis factor-induced optic neuropathy. Invest Ophthalmol Vis Sci.

[34] Sompol P, Norris CM (2018). Ca^2+^, Astrocyte activation and calcineurin/NFAT signaling in age-related neurodegenerative diseases. Front Aging Neurosci.

