# Supplementary figures and images for: Axonal Protection by Tacrolimus with Inhibition of NFATc1 in TNF-Induced Optic Nerve Degeneration
Source: Neurochem Res. 2019 May 13;44(7):1726–35. doi: 10.1007/s11064-019-02804-6 (PMC6555779; doi:10.1007/s11064-019-02804-6)

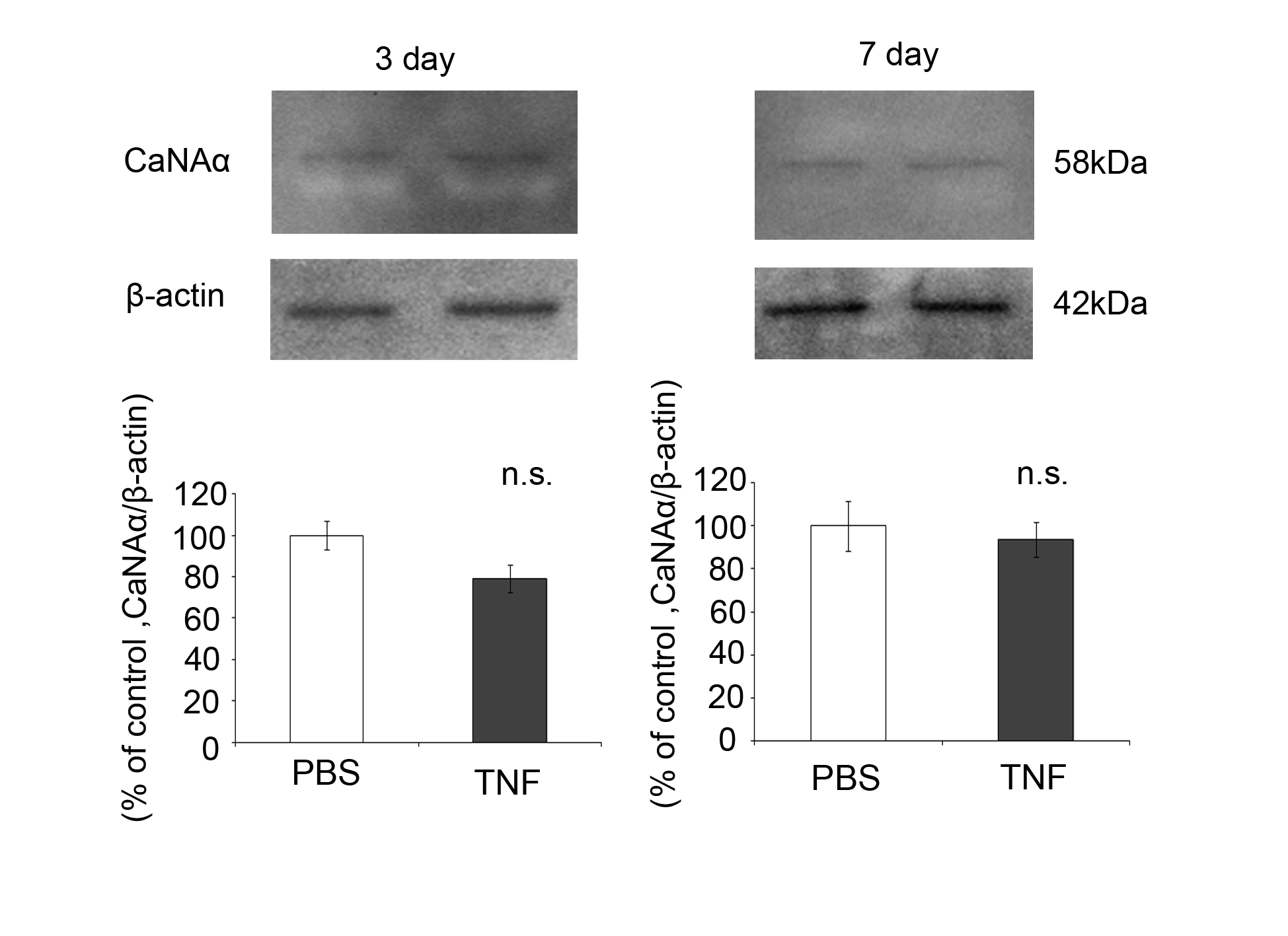

Supplement: Supplementary file 1 — Supplementary material 1 (TIFF 2709 kb) [file 11064_2019_2804_MOESM1_ESM.tif]

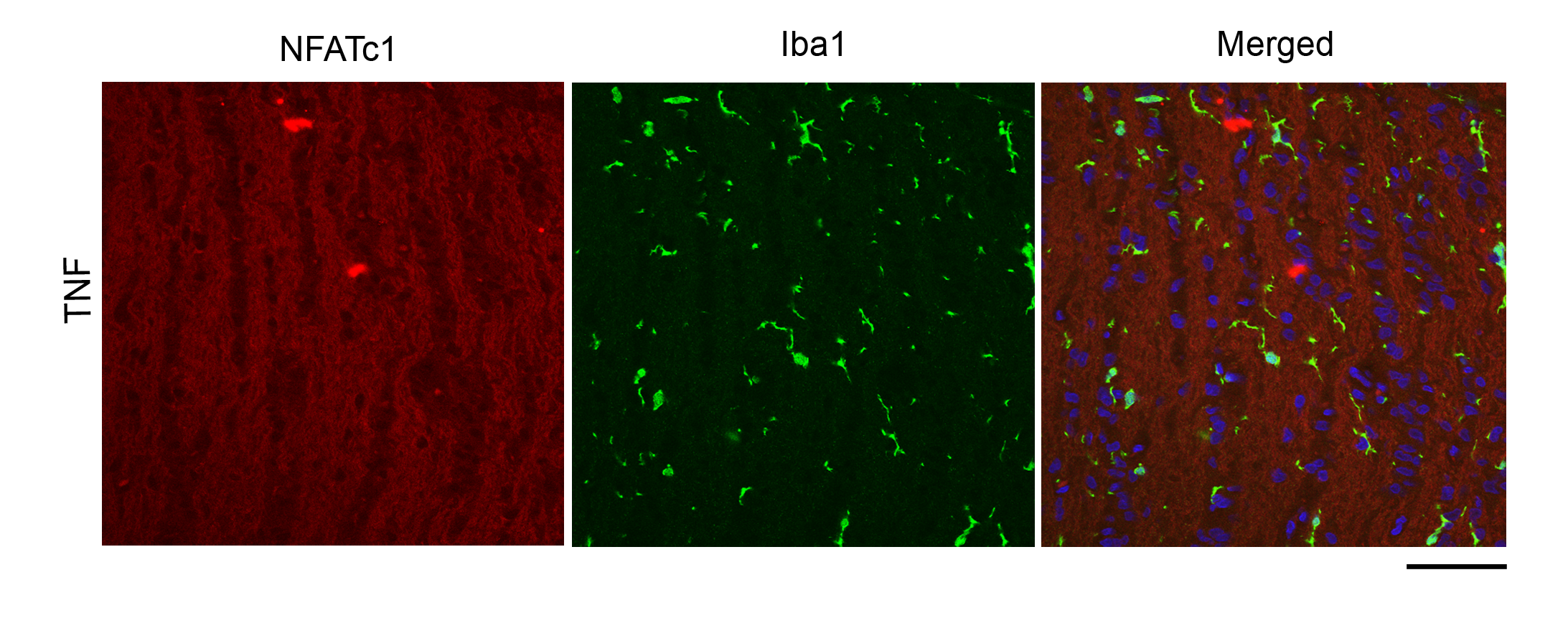

Supplement: Supplementary file 2 — Supplementary material 2 (TIFF 5688 kb) [file 11064_2019_2804_MOESM2_ESM.tif]
